# *Luteirhabdus pelagi* gen. nov., sp. nov., a novel member of the family *Flavobacteriaceae*, isolated from the West Pacific Ocean

**DOI:** 10.1007/s00203-021-02557-3

**Published:** 2021-10-26

**Authors:** Wen-Ting Ren, Fan-Xu Meng, Li-Li Guo, Li Sun, Xue-Wei Xu, Peng Zhou, Yue-Hong Wu

**Affiliations:** 1 Key Laboratory of Marine Ecosystem Dynamics, Ministry of Natural Resources & Second Institute of Oceanography, Ministry of Natural Resources, Hangzhou, 310012 People’s Republic of China; 2 School of Oceanography, Shanghai Jiao Tong University, Shanghai, 200240 People’s Republic of China; 3 College of Life and Environmental Science, Hunan University of Arts and Science, Changde, 415000 People’s Republic of China; 4 State Research Center of Island Exploitation and Management, Ministry of Natural Resources & Second Institute of Oceanography, Ministry of Natural Resources, Hangzhou, 310012 People’s Republic of China

**Keywords:** *Luteirhabdus*, *Flavobacteriaceae*, *Bacteroidetes*, Phylogenetic analysis, West Pacific Ocean, Genomic analysis

## Abstract

A Gram-stain-negative, aerobic, and yellow-pigmented bacterium, designated A3-108^T^, was isolated from seawater of the West Pacific Ocean. Cells were non-motile and rod-shaped, with carotenoid-type pigments. Strain A3-108^T^ grew at pH 6.0–8.5 (optimum 6.5) and 15–40 °C (optimum 28 °C), in the presence of 0.5–10% (w/v) NaCl (optimum 1.0%). It possessed the ability to produce H_2_S. Based on the 16S rRNA gene analysis, strain A3-108^T^ exhibited highest similarity with *Aureisphaera salina* A6D-50^T^ (90.6%). Phylogenetic analysis shown that strain A3-108^T^ affiliated with members of the family *Flavobacteriaceae* and represented an independent lineage. The principal fatty acids were iso-C_15:0_, iso-C_17:0_ 3-OH, iso-C_15:1_ G, and summed feature 3 (C_16:1_*ω*7*c* and/or C_16:1_*ω*6*c*). The sole isoprenoid quinone was MK-6. The major polar lipids were phosphatidylethanolamine, one unidentified aminophospholipid, one unidentified aminolipid and one unidentified lipid. The ANIb, in silico DDH and AAI values among the genomes of strain A3-108^T^ and three reference strains were 67.3–71.1%, 18.7–22.1%, and 58.8–71.4%, respectively. The G + C content was 41.0%. Distinctness of the phylogenetic position as well as differentiating chemotaxonomic and other phenotypic traits revealed that strain A3-108^T^ represented a novel genus and species of the family *Flavobacteriaceae*, for which the name *Luteirhabdus pelagi* gen. nov., sp. nov. is proposed (type strain, A3-108^T^ = CGMCC 1.18821^T^ = KCTC 82563^T^).

**Supplementary Information:**

The online version contains supplementary material available at 10.1007/s00203-021-02557-3.

## Introduction

The family *Flavobacteriaceae,* belonging to the class *Flavobacteriia*, the phylum *Bacteroidetes*, was first proposed by Jooste (1985), validly published by Reichenbach (1992), then emended by Bernardet et al. (1996, 2002) and García-López et al. (2019). Prior to 2021, the family *Flavobacteriaceae* comprised 150 genera with validly published names. This study focuses on the description of a novel genus and species with the type strain A3-108^T^ was isolated from seawater collected at the seamount area in the West Pacific Ocean.

Seamounts are defined as the huge uplifts located below sea level, and exceed 1000 m in height, and are unique environments widely distributed on the deep-ocean sub-seafloor (Yesson et al. 2011). The West Pacific Ocean has the most concentrated area of global seamount systems (Qin and Yin 2011). Seamounts are important habitats for marine organisms (Clark et al. 2010). In the upper water column, primary productivity is influenced by topographically induced turbulent mixing in the seamounts' ecosystem (Boehlert and Genin 1987; Polzin et al. 1997), which has a strong impact on physical/chemical parameters and organism communities (Mashayek et al. 2017; Muck et al. 2014). Currently, there are few studies on microbial communities in seamount environments.

## Materials and methods

### Samples and isolation

During the investigation of bacterial diversity, a seawater sample from the seamount area in the West Pacific Ocean (at a depth of 300 m, 23.2° N, 162.3° E), was collected by a rosette sampler connected with CTD system (SBE911 plus; Sea-Bird Electronics, Inc. USA) in 2018. Aboard the ship, the seawater sample was subjected to the culture process immediately. Approximately 100 µL seawater samples were diluted using serial dilution technique and added to different media. The strain A3-108^T^ was isolated aerobically on natural seawater agar (1 L filtered natural seawater supplemented with 0.5 g peptone (BD Difco), 0.1 g yeast extract (BD Difco), 20 g agar (BD Difco), pH 7.2–7.4) and purified by repeated restreaking. The purity was confirmed by the uniformity of cell morphology. Unless otherwise stated, strain A3-108^T^ was routinely cultured in marine broth 2216 (MB, BD Difco) or on marine agar 2216 (MA, BD Difco) at 30 °C and maintained at − 80 °C with 30% (v/v) glycerol. *Galbibacter mesophilus* CGMCC 1.15663^T^ and *Marixanthomonas ophiurae* JCM 14121^T^, were obtained from the CGMCC (China General Microbiological Culture Collection Center) and JCM (Japan Collection of Microorganisms), respectively. An additional reference strain *Marinirhabdus gelatinilytica* NH83^T^ was obtained from our laboratory (Wu et al. 2016).

### 16S rRNA gene and genome sequence determination

High-quality genomic DNA was extracted by Nucleic Acid Purification kit (Dongsheng Biotech). The 16S rRNA gene was amplified by the universal primers 27F/1492R (27F: 5'-AGAGTTTGATCCTGGCTCAG-3'; 1492R: 5'-GGYTACCTTGTTACGACTT-3'). The PCR thermal cycling conditions were as follows: 30 cycles of 98 °C for 10 s, 55 °C for 10 s, and 72 °C for 30 s. The PCR products were purified and sequenced by Sanger sequencing to obtain the almost complete 16S rRNA gene sequence.

The genomic DNA of strain A3-108^T^ and *Galbibacter mesophilus* CGMCC 1.15663^T^ were sequenced by Solexa paired-end sequencing technology with the Illumina NovaSeq 6000 PE150 platform (Novogene Co. Ltd, Tianjing). One paired-end library was constructed with insert size of 350 bp. The sequencing generated approx. 1 Gb clean data (approx. 500-fold genome coverage). De novo assembly of the reads was carried out using SOAPdenovo (version 2.0.1) (Luo et al. 2012). The completeness of genome sequences was addressed using the bioinformatics tool CheckM (http://ecogenomics.github.io/CheckM/) (Parks et al. 2015). The complete 16S rRNA gene was annotated via the RNAmmer 1.2 Server (Lagesen et al. 2007) and compared with gene sequences obtained from PCR to ensure its authenticity.

### Phylogenetic status and DNA relatedness

The 16S rRNA gene sequence was compared with the corresponding sequences of closely related organisms via online EzBioCloud service (https://www.ezbiocloud.net) (Yoon et al. 2017). Based on 16S rRNA gene similarity, 23 species were selected and aligned for phylogenetic analysis by CLUSTALW software (Thomson et al. 1994). Phylogenetic trees were constructed using MEGA 7.0 program package (Kumar et al. 2016) using the methods of neighbor-joining (Saitou and Nei 1987), maximum-parsimony (Fitch 1971) and maximum-likelihood (Felsenstein 1981). Evolutionary distances of the neighbor-joining method were calculated according to the Kimura-2-parameter algorithm model (Kimura 1980).

A phylogenomic tree was constructed based on single-copy orthologous clusters (OCs) of strain A3-108^T^ and its related taxa of the family *Flavobacteriaceae*. The related genome sequences were obtained from the NCBI GenBank database and annotated using the Prokka server (Seemann 2014). Orthologous clusters (OCs) were selected by Proteinortho (version 5.16b) (Lechner et al. 2014). Single-copy OCs were filtered by an in-house shell script. Protein sequences were aligned using MAFFT (version 7) (Katoh and Standley 2013). Aligned sequences were refined via trimAL (version 1.4.1) (Capella-Gutiérrez et al. 2009) and concatenated by an in-house shell script. The best substitution model was estimated by IQ-Tree software (version 1.6.1) (Nguyen et al. 2015) and the model LG + F + R4 was selected. The maximum-likelihood phylogenomic tree was reconstructed through IQ-Tree software and visualized applying MEGA 7.0 software (Kumar et al. 2016).

The average nucleotide identity (ANI) values, the DNA–DNA hybridization (DDH) values and the average amino acid identity (AAI) values were calculated using JSpeciesWS (http://jspecies.ribohost.com/jspeciesws/), Genome-to-Genome Distance Calculator (GGDC; version 2.1) (https://ggdc.dsmz.de/home.php) and AAI calculator (http://enve-omics.ce.gatech.edu/aai/), respectively (Richter et al. 2016; Meier-Kolthoff et al. 2013; Luis et al. 2014). Orthologous average nucleotide identity (OrthoANI) values were calculated by OAT (Chun et al. 2016).

### Phenotypic characteristics

Cell morphology, ultrastructure, size, and the presence of flagellum were observed by transmission electron micrographs (JEM-1230, JEOL). Gram reaction was determined by the Gram-Stain method (Brown and Hopps 1973). Motility was examined by stab culture with semi-solid medium, using MB supplemented with 0.5% (w/v) agar (Wolfe and Berg 1989). The temperature range for growth was investigated by incubating in MB at 4, 15, 20, 28, 30, 37, 40, 45, and 50 °C. The pH range for growth was determined in MB with different pH (pH 5.0–10.5, in 0.5 pH unit intervals) using appropriate biological buffers at 50 mM concentration (MES for pH 5.0–6.0, PIPES for pH 6.5–7.0, Tricine for pH 7.5–8.5, CAPSO for pH 9.0–10.0 and CAPS for pH 10.5). The optimal conditions with NaCl for growth were measured using NaCl-free MB (prepared according to the MB formula, but without NaCl) with different NaCl concentrations (0, 0.5, 1.0, 3.0, 5.0, 7.5, 10.0, 15.0, 20.0, and 25%, w/v). Cell densities were monitored by measuring with a UV/Visible Spectrophotometer at 600 nm (Ultrospec 6300 pro; Amersham Biosciences). Anaerobic growth was tested by the Anaero-Pack (Mitsubishi) adding sodium nitrate (10 mM), sodium sulfate (10 mM), and sodium thiosulfate (10 mM) as potential electron acceptors on the MA. The growth curve of strain A3-108^T^ was determined by incubation in MB with the optimal growth condition (30 °C, 180 rpm), and cell densities were measured every 2 h incubation via measuring OD_600_ in a UV/Visible Spectrophotometer (Genesys 50; Thermofisher Scientific). The doubling time and the specific growth rate were calculated by formulas:

doubling time (*t*_*d*_/h) = ln2/*k*; specific growth rate (*μ*/h^−1^) = 1/*t*_*d*_;

*k* represents relative growth rate (slope of the curve) (Monod 1949).

Flexirubin-type pigments were detected by a bathochromic shift test (Fautz and Reichenbach 1980). Carotenoid-type pigments were detected by pigment absorption spectrum analysis as described by Hildebrand et al. (1994). Pigments were extracted with acetone/methanol (7:2, v/v) and performed by a Beckman DU 800 Spectrophotometer (detection wavelength from 300 to 800 nm).

Oxidase and catalase activities, H_2_S production from sodium thiosulfate (0.5%, w/v), sodium sulfate (0.5%, w/v), and cysteine (0.5%, w/v) and the hydrolysis abilities of starch (0.2%, w/v), DNA ((0.2%, w/v)), L-tyrosine (0.5%, w/v), esculin (0.1%, w/v), CM-cellulose (1.0%, w/v), and Tween 40 (1.0%, w/v), 60 (1.0%, w/v), and 80 (1.0%, w/v) were determined as previously described (Dong and Cai 2001). Acid production was examined by MOF medium supplemented with 0.5% alcohols or sugars (Leifson 1963). The activities of enzymes, including nitrate reduction and assimilation carbohydrates, were tested by API ZYM and API 20NE tests (bioMérieux) at 30 °C. API ZYM strips were read after 24 h and API 20NE strips were read after 48 h, according to the manufacturer's instructions. Three reference strains, *Marinirhabdus gelatinilytica* NH83^T^, *Galbibacter mesophilus* CGMCC 1.15663^T^ and *Marixanthomonas ophiurae* JCM 14121^T^, were used as controls in the above tests.

### Chemotaxonomic analysis

The cellular fatty acids of the strain A3-108^T^ and three reference strains were determined under identical conditions in parallel. Approximately 20 mg of cells were harvested by the quadrant streak method on MA plates at 30 °C for 3 days (quadrant 3 exhibiting confluent growth). Fatty acids were extracted by saponification, methylation, and extraction as described previously (Sasser 1990). The cellular fatty acids were analyzed by 6890 gas chromatograph according to Microbial Identification System (MIDI).

For polar lipids and isoprenoid quinones analyses, strain A3-108^T^ was cultivated in MB at 30 °C for 3 days to obtain cell biomass. Respiratory quinones were extracted from cells (around 200 mg) with chloroform/methanol (2:1, v/v) and analyzed by LC–MS (Agilent) (Komagata and Susuki 1987). Polar lipids were extracted and separated by two-dimensional TLC (Tindall et al. 2007), with chloroform/methanol/water (13:5:0.8, v/v) for the first direction and chloroform/methanol/acetic/water (16:2.4:3:0.8, v/v) for the second direction. Total lipids, aminolipids, phospholipids, and glycolipids were detected by molybdatophosphoric acid, 0.5% ninhydrin reagent, Molybdenum Blue spray reagent (SIGMA), and 0.5% α-naphthol reagent with methanol/water (1:1, v/v), respectively (Komagata and Susuki 1987).

### Genomic analysis

The draft genome sequence was annotated using the RAST server online (https://rast.nmpdr.org/rast.cgi) (Aziz et al. 2008), and annotation information including predicted coding sequences (CDSs), proteins and RNAs were obtained. Metabolic pathways were predicted using the Kyoto Encyclopedia of Genes and Genomes (KEGG) online annotation server (Kanehisa et al. 2016).

## Results and discussion

### 16S rRNA gene sequence similarities and phylogenetic analysis

The almost complete 16S rRNA gene sequence of strain A3-108^T^ was obtained. According to the results of EzBioCloud, the strain A3-108^T^ was closely related to members of the family *Flavobacteriaceae* and its 16S rRNA gene sequence showed the highest similarity to *Aureisphaera salina* A6D-50^T^ (90.6%), followed by *Galbibacter mesophilus* Mok-17^T^ (90.5%), *Marinirhabdus gelatinilytica* NH83^T^ (90.4%), *Aureisphaera galaxeae* 04OKA003-7^T^ (90.4%) and *Aequorivita aestuarii* JC2436^T^ (90.2%).

**Fig. 1:**
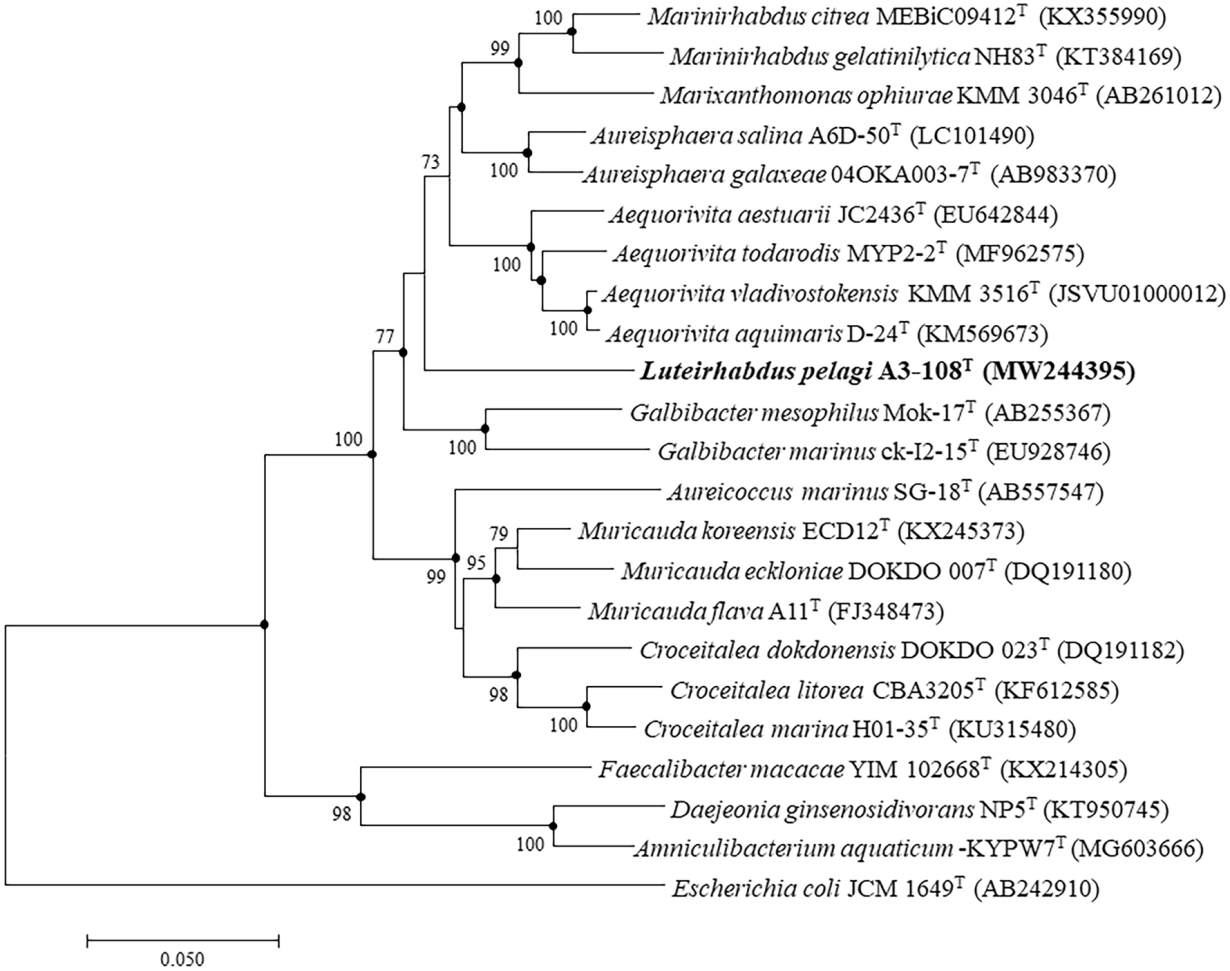
Neighbor-joining phylogenetic tree based on 16S rRNA gene sequences showing the phylogenetic relationships of the strain A3-108^T^ and related taxa. Bootstrap values (expressed as percentages of 1000 replications) of 70% or more are shown at branch nodes. Filled circles indicate that the corresponding nodes were also recovered in the trees generated with the maximum-likelihood and maximum-parsimony algorithms. Bar, 0.05 substitutions per nucleotide position

**Fig. 2:**
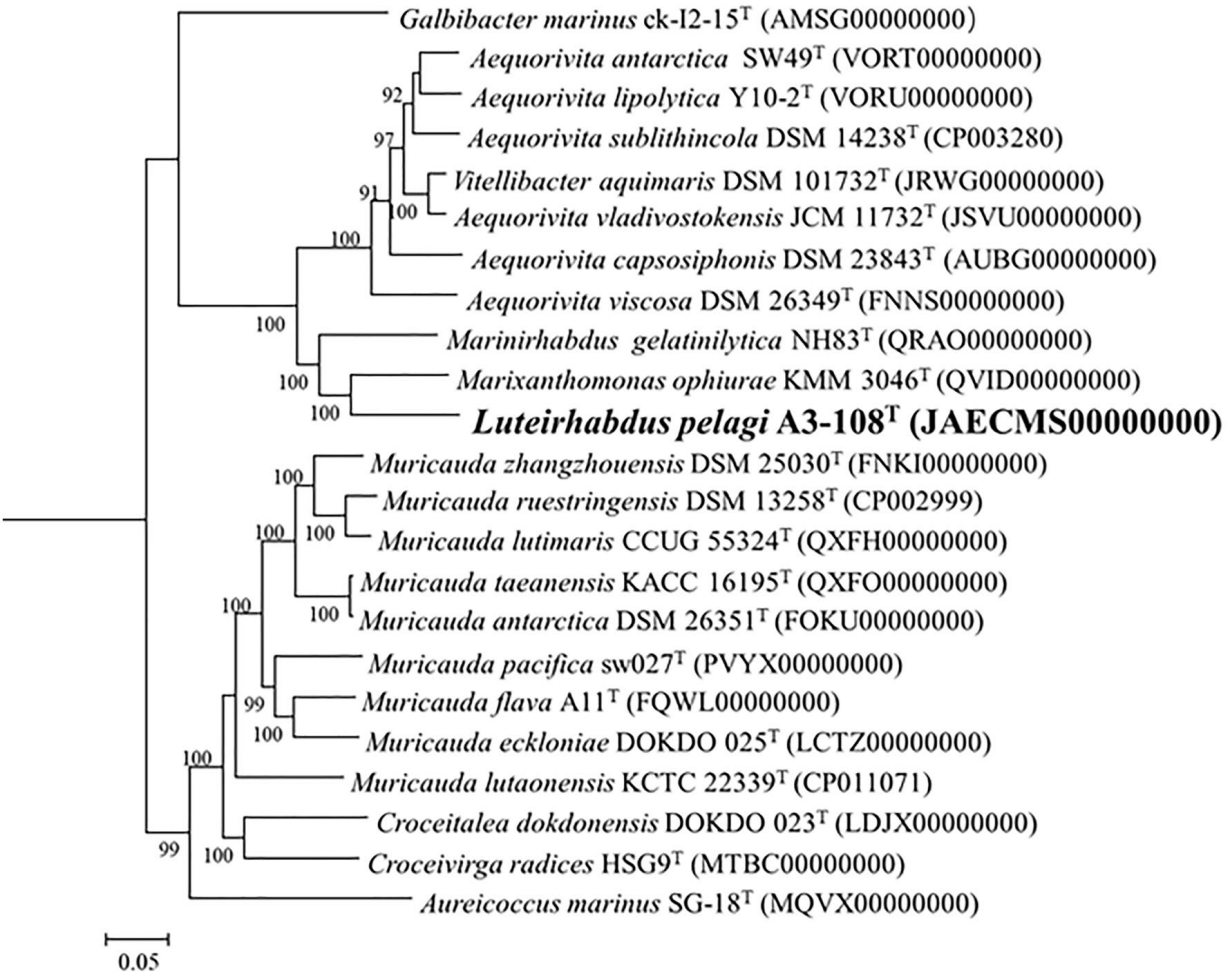
Phylogenomic tree based on the genomic sequences reflecting the phylogenetic relationship of the strain A3-108^T^ and the related taxa. Bootstrap values (> 90%) based on 100 replications are shown at brand nodes. *Escherichia coli* K-12^T^ (GenBank assembly accession number is GCA_000005845.2) was used as outgroup (not shown in the phylogenetic tree). Bar, 0.05 substitutions per genomic sequence position

The phylogenetic trees manifested that strain A3-108^T^ fall into the family *Flavobacteriaceae* and formed a separated branch apart from other genera of the family with high bootstrap values (Fig. 1). Phylogenetic analysis indicated that the strain A3-108^T^ represented an independent lineage in family *Flavobacteriaceae*. Furthermore, the maximum-likelihood phylogenomic tree based on single-copy orthologous clusters (OCs) demonstrated that strain A3-108^T^ affiliated with the family *Flavobacteriaceae* and clustered with *Marixanthomonas ophiurae* KMM 3046^T^ (Fig. 2).

### Genomic features and DNA–DNA relatedness

Based on the bioinformatic tool CheckM, the genome completeness of strain A3-108^T^ was 99.2%, with 0.27% contamination. The genome sequence estimated to be ≥ 95% completeness, with ≤ 5% contamination, was considered to be an excellent reference genome for deeper analyses (Pruesse et al. 2007). The final genome of strain A3-108^T^ comprised a total size of 3.40 Mb with 99 contigs, and G + C content was 41.0%. The assembled scaffolds annotated by RAST online, harbored a total of 3250 coding genes, 37 tRNAs and 5 rRNAs. The general genomic features of strain A3-108^T^ and reference strains are shown in Supplementary Table S1. The ANIb and in silico DDH among the genome of strain A3-108^T^ and the reference strains were 67.3–71.1% and 18.7–22.1%, respectively (Supplementary Table S2). The ANI values were far below the species threshold of 94–96% (Richter and Rosselló-Móra 2009) and the genus demarcation boundary of 90% (Barco et al. 2020). The in silico DDH values were below the threshold value 70% that corresponded to the species boundary (Wayne et al. 1987). In addition, the AAI values were 58.8–71.4% among the genome of strain A3-108^T^ and the reference strains (Supplementary Table S2), which were below the species cutoff 95–96% (Konstantinidis and Tiedje 2005) and the threshold of 60–80% to distinguish genera from each other (Luo et al. 2014). The OrthoANI values between strain A3-108^T^ and the reference strains were 67.7–71.5% (Supplementary Table S2). The ANI, in silico DDH and AAI values indicted a low taxonomic relatedness between strain A3-108^T^ and the reference strains of the family *Flavobacteriaceae*.

### Phenotypic features

Strain A3-108^T^ was Gram-stain-negative, aerobic, non-motile, and rod-shaped with 0.5–0.8 μm in width and 1.6–3.9 μm in length (Supplementary Fig. S1). No flagellum was observed. Colonies were yellow, circular, convex, opaque, smooth, and 1–2 mm in diameter after 3 days of incubation at 30 °C on MA. The growth range of pH, temperature and NaCl concentrations were pH 6.0–8.5, 15–40 °C, and 0.5–10% (w/v) on MB. The growth of strain A3-108^T^ contained three phases, including the lag phase (0–16 h), exponential phase (16–40 h), and stationary phase (exceed 40 h) at 30 °C on MB. The doubling time and the specific growth rate were 6.2 h and 0.16 h^−1^, respectively (Supplementary Fig. S2). Strain A3-108^T^ was positive for oxidase, arginine dihydrolase, nitrite reduction, and H_2_S production. Carotenoid-type pigments were present but flexirubin-type pigments were not (Supplementary Fig. S3). Additional phenotypic properties are given in the species description, Tables 1, 3 and Supplementary Table S3.

**Table 1:** Differential phenotypic characteristics among strain A3-108^T^ and its related taxa

Characteristics	1	2	3	4
Growth in NaCl (%, w/v):				
Range	0.5–10	0.5–7.5*	3.0–7.0^†^	1.0–12.0^‡^
Optimum	1.0	2.0–5.0*	3.0–5.0^†^	3.0–5.0^‡^
Growth in pH:				
Range	6.0–8.5	6.5–7.5*	−^†^	−^‡^
Optimum	6.5	7.0*	−^†^	6.5–8.5^‡^
Growth temperature (°C):				
Range	15–40	4–37*	10–42^†^	5–32^‡^
Optimum	28	30*	25–30^†^	25–28^‡^
Nitrate reduction	−	−	+	−
Hydrolysis of				
Catalase	−	+	+	+
Esculin	−	−	+	−
L-Tryosine	−	−	+	−
Tween 60	−	−	+	+
API ZYM system	−			
* N*-Acetyl-*β-*glucosaminidase	−	−	+	−
* α*-Galactosidase	−	−	+	−
* β*-Galactosidase	−	−	+	−
* α*-Glucosidase	−	−	+	−
* β*-Glucosidase	−	−	+	−
API 20NE system				
Arginine dihydrolase	+	−	−	−
Assimilation D-glucose	−	−	+	−
Assimilation D-mannose	−	−	+	−
Assimilation D-maltose	−	−	+	−
Hydrolysis (*β*-glucosidase) esculin	−	−	+	−
* β*-Galactosidase (para-nitrophenyl-*β-*D-galactopyranosidase)	−	−	+	−
Acid production from				
D-Cellobiose	−	−	+	−
D-Galactose	−	−	+	−
D-Maltose	−	−	+	−
D-Salicin	−	−	+	−
D-Trehalose	−	−	+	−
D-Xylose	−	−	+	−
H_2_S production	+	+	−	+

Strains/species: 1, strain A3-108^T^; 2, *Marinirhabdus gelatinilytica* NH83^T^; 3, *Galbibacter mesophilus* CGMCC 1.15663^T^; 4, *Marixanthomonas ophiurae* JCM 14121^T^. All data were obtained from this study unless indicated. +  positive; − negative

*Data were taken from Wu et al. (2016)

^†^Data were taken from Shams et al. (2007)

^‡^Data were taken from Romanenko et al. (2007)

### Chemotaxonomic analysis

The sole respiratory quinone detected in strain A3-108^T^ was menaquinone-6 (MK-6). Strain A3-108^T^ possessed phosphatidylethanolamine (PE), one unidentified aminophospholipid (APL), one unidentified aminolipid (AL) and one unidentified lipid (L1) as major polar lipids. In addition, two unidentified aminoglycolipids (AGL1-2), one unidentified glycolipid (GL), and two unidentified lipids (L2-3) were present as moderate or minor lipids (Supplementary Fig. S4). The major fatty acids of strain A3-108^T^ contained iso-C_15:0_ (22.4%), iso-C_17:0_ 3-OH (17.2%), iso-C_15:1_ G (15.7%), and summed feature 3 (C_16:1_*ω*7*c* and/or C_16:1_*ω*6*c*) (10.3%) (Table 2).

**Table 2:** Fatty acid composition (%) of strain A3-108^T^ and its related taxa

Fatty acid	1	2	3	4
Straight-chain				
C_16:0_	2.5	tr	1.1	4.6
Unsaturated				
C_15:1_*ω*6*c*	–	–	2.4	1.4
C_17:1_*ω*6*c*	–	–	1.2	–
C_17:1_*ω*8*c*	–	–	tr	tr
iso-C_15:1_ G	**15.7**	7.3	7.8	3.2
iso-C_16:1_ G	1.3	tr	–	–
Hydroxy				
C_15:0_ 2-OH	1.7	tr	1.3	1.2
C_15:0_ 3-OH	-	tr	2.7	–
iso-C_15:0_ 3-OH	3.1	6.1	**10.3**	3.3
iso-C_16:0_ 3-OH	4.5	6.3	2.0	8.6
C_16:0_ 3-OH	tr	tr	tr	1.1
C_17:0_ 2-OH	5.7	1.0	tr	2.3
iso-C_17:0_ 3-OH	**17.2**	**26.3**	**28.1**	**17.3**
Branched-chain				
iso-C_13:0_	–	1.3	tr	–
iso-C_15:0_	**22.4**	**35.8**	**11.5**	**22.0**
anteiso-C_15:0_	9.2	2.7	tr	3.2
anteiso-C_15:1_ A	2.3	–	–	–
iso-C_16:0_	1.9	2.9	tr	8.7
iso-C_16:1_ H	–	–	1.2	3.5
Summed feature*				
3	**10.3**	6.2	**16.8**	**12.3**
9	–	–	8.5	4.6

Strains/species: 1, strain A3-108^T^; 2, *Marinirhabdus gelatinilytica* NH83^T^; 3, *Galbibacter mesophilus* CGMCC 1.15663^T^; 4, *Marixanthomonas ophiurae* JCM 14121^T^. All data were obtained from this study. Fatty acids representing less than 1.0% in all strains were omitted and the amounts > 10% were in bold. − Not detected, *tr* traces (< 1.0%)

*Summed features represent groups of two fatty acids that could not be separated by GLC with the MIDI system. Summed feature 3 contained C_16:1_*ω*7*c* and/or C_16:1_*ω*6*c*; Summed features 9 contained C_16:0_ 10-methyl and/or iso-C_17:1_*ω*9*c*

Chemotaxonomic analysis supported the result of the phylogenetic analysis. The sole respiratory quinone detected in strain A3-108^T^ was consistent with members of the family *Flavobacteriaceae* (Bernardet 2015). The components iso-C_17:0_ 3-OH and iso-C_15:0_ were major fatty acids in strain A3-108^T^ and the reference strains (Table 2). The presence of phosphatidylethanolamine (PE) was conserved in strain A3-108^T^ and the related genera (Table 3).

**Table 3:** Differential properties of strain A3-108^T^ and its adjacent genera

Characteristics	1	2	3	4	5	6
Motility^‡^	NM	NM	NM	NM	NM	NM, GM
Colony colour*	Y	Y, LY	Y	Y	Y, PY	Y, BY, YO, O, VOY
Shape^†^	R	R	R	R	R, C	R, O, F
Temperature range (°C)	15–40	4–38	10–42	ND	20–37	-2–43
Optimum temperature (°C)	28	30	25–32	5–32	28–30	20–37
NaCl range (%)	0.5–10	0.5–7.5	0–9	3.0–5.0	0.5–5.5	0–12
Optimum NaCl (%)	1.0	2.0–5.0	1.0–5.0	1–12	3	1–6
pH range	6.0–8.5	4.0–8.5	NA	6.5–8.5	7.0–9.0	5.0–10.0
Optimum pH	6.5	7.0	NA	NA	7.0	6.0–9.0
Catalase	−	+	+	+	v	v
Oxidase	+	−	+	+	−	v
Hydrolysis of						v
CM-cellulose	−	NA	−	−	NA	−
Esculin	−	+	+	−	NA	v
Gelatin	+	+	+	+	+	v
L-Tryosine	−	NA	NA	NA	NA	v
Starch	−	−	+	−	−	v
Tween 60	−	+	NA	NA	NA	v
Nitrate reduction	−	+	+	−	−	v
H_2_S production	+	−	NA	−	NA	v
Major fatty acids^§^	I15:1G, I17:0O, I15:0, SF3	I15:0, I15:1G, A15:0, I16:0, I17:0O, SF3	I15:0, I15:1, I17:0O, SF3, SF9	I16:0O, I17:0O, A17:0O	I17:0O, I15:0, I15:1G, SF3	I15:0, I17:0O, A15:0, I15:1G, I17:1*ω*9*c*, SF3, I15:1*ω*10*c*, A15:1*ω*10*c*, I16:1*ω*6*c*, I17:1*ω*5*c*, I16:0O
Major polar lipids^¶^	PE, APL, AL, L	PE, APL, AL, L	AL, PE, L	APL, PE, LPE, SL, L	PE, AL, L	PE, L, AL, PL, PGL
DNA G + C content (% or mol%)	41.0	41.0–43.1	37–38	37.3	40.8–41.0	33.0–48.7

Strains/species: 1, strain A3-108^T^ (this study); 2, *Marinirhabdus* (Wu et al. 2016; Yang et al. 2018); 3, *Galbibacter* (Li et al. 2013; Shams et al. 2007); 4, *Marixanthomonas* (Romanenko et al. 2007); 5, *Aureisphaera* (Yoon et al. 2015, 2016); 6, *Aequorivita* (Bowman 2002; Kim et al. 2010, 2018; Lin et al. 2015; Liu et al. 2013; Nedashkovskaya et al. 2003; Park et al. 2009, 2014; Rajasabapathy et al. 2015; Thevarajoo et al. 2016; Wang et al. 2020; Zhang et al. 2020). +  positive, − negative, *v* positive or negative, *NA* no data available

^‡^*NM* non-motile, *GM* gliding motility

*Y yellow, *LY* lemon-yellow, *PY* pale-yellow, *BY* bright-yellow, *YO* yellow-orange, *O* orange, *VOY* vivid orange yellow

^†^*R* rod, *C* coccus, *O* ovoid, *F* filaments

^§^*SF3* Summed feature 3, *SF9* Summed feature 9, *I15:0* iso-C_15:0_, *I15:1G* iso-C_15:1_ G, *I17:0O* iso-C_17:0_ 3-OH, *I16:0* iso-C_16: 0_, *I15:1* iso-C_15: 1_, *I16:0O* iso-C_16: 0_ 3-OH, *A17:0O* anteiso-C_17: 0_ 3-OH, *16:0* C_16:0_, *18:0* C_18:0_, *A15:0* anteiso-C_15:0_, *I15:0* iso-C_15:0_, *I17:1ω9c* iso-C_17: 1_*ω*9*c*, *I17:1ω5c* iso-C17: 1*ω*5*c*, *I15:1ω10c* iso-C_15: 1_*ω*10*c*, *A15:1ω10c* anteiso-C_15: 1_*ω*10*c*, *I16:1ω6c* iso-C_16: 1_*ω*6*c*

^¶^*PE* phosphatidylethanolamine, *APL* aminophospholipid, *AL* aminolipid, *L* lipid, *LPE* lysophosphatidylethanolamine, *SL* sphingolipid, *PL* phospholipid, *PGL* phosphoglycolipid

The chemotaxonomic results also showed some differences clearly in fatty acid compositions and polar lipid profiles between strain A3-108^T^ and the reference strains. The component iso-C_15:1_ G was presented as major fatty acid in the strain A3-108^T^ (15.7%), while it was presented as moderate fatty acid in *Marinirhabdus gelatinilytica* NH83^T^, *Galbibacter mesophilus* CGMCC 1.15663^T^ and *Marixanthomonas ophiurae* JCM 14121^T^ (3.2–7.8%). In addition, the component anteiso-C_15:1_ A was only detected in the strain A3-108^T^. In addition, the fatty acid of strain A3-108^T^ were different from the reference strains in the compositions and proportions (Table 2). With respect to polar lipid profiles, the component of the unidentified aminophospholipid (APL) was presented as major polar lipid in the strain A3-108^T^, while it was not presented in *Galbibacter mesophilus* CGMCC 1.15663^T^. Besides, the unidentified aminolipid (AL), one of the major polar lipids, was not presented in the related strain *Marixanthomonas ophiurae* JCM 14121^T^. In addition, moderate polar lipids, including an unidentified glycolipid (GL) and two aminoglycolipids (AGL1-2), were presented in the strain A3-108^T^, while they were not detected in the reference strains. Besides, lysophosphatidylethanolamine (LPE) and sphingolipid (SL) were only detected in *Marixanthomonas ophiurae* JCM 14121^T^ (Supplementary Fig. S4 and Wu et al. 2016; Romanenko et al. 2007; Hameed et al. 2014).

## Conclusion

Phylogenetic analysis indicated that the strain A3-108^T^ represented an independent lineage in family *Flavobacteriaceae*. Strain A3-108^T^ could be distinguished from the related genera and type strains of the family *Flavobacteriaceae* by phenotypic characteristics differences such as the range and optimum for growth of NaCl, pH and temperature, enzyme activities, assimilation carbohydrates and acid production (Tables 1, 3). Based on the phylogenetic analysis, physiological, and chemotaxonomic characteristics, as well as genome analysis, strain A3-108^T^ represents a novel genus and species in the family *Flavobacteriaceae*, for which the name *Luteirhabdus pelagi* gen. nov., sp. nov. is proposed.

### Description of *Luteirhabdus* gen. nov.

*Luteirhabdus* (Lu.te.i.rhab'dus. L. masc. adj. *luteus* yellow; Gr. fem. n. *rhabdos* rod; N.L. fem. n. *Luteirhabdus* a yellow rod-shaped bacterium).

Cells are Gram-stain-negative, strictly aerobic, non-motile and rod-shaped. No flagellum was observed. Carotenoid-type pigments are produced. Positive for oxidase, H_2_S production. The predominant menaquinone is MK-6. Major polar lipids are phosphatidylethanolamine, one unidentified aminophospholipid, one unidentified aminolipid, and one unidentified lipid. The major cellular fatty acids are iso-C_15:0_, iso-C_17:0_ 3-OH, iso-C_15:1_ G, and summed feature 3 (C_16:1_*ω*7*c* and/or C_16:1_*ω*6*c*). The DNA G + C content of the type species is 41.0%. The genus belongs to the family *Flavobacteriaceae*, class *Flavobacteriia*, phylum *Bacteroidetes*. The type species is *Luteirhabdus pelagi*.

### Description of *Luteirhabdus pelagi* sp. nov.

*Luteirhabdus pelagi* (pe.la'gi. L. gen. n. *pelagi* of the open sea).

Cells are Gram-stain-negative, strictly aerobic, non-motile, and rod-shaped with 0.5–0.8 μm in width and 1.6–3.9 μm in length. Colonies are yellow, circular, convex, opaque, smooth, and 1–2 mm in diameter after 3 days of incubation at 30 °C on MA. Requires Na^+^ ions for growth. Growth occurs in NaCl-free MB supplement with 0.5–10% (w/v) NaCl (optimum at 1.0%). The pH and temperature ranges for growth are pH 6.0–8.5 and 15–40 °C (optimum at pH 6.5 and 28 °C). Carotenoid-type pigments are produced but flexirubin-type pigments are not. No anaerobic growth occurs on MA supplemented with sodium nitrate, sodium sulfate, and sodium thiosulfate. Positive for oxidase, arginine dihydrolase, gelatin, and nitrite reduction. Negative for catalase, indole production, glucose fermentation, urease, and nitrate reduction. Negative for the degradation of Tweens 40, Tweens 60, L-Tryosine, starch, esculin, CM-cellulose, DNA, and *β*-galactosidase. H_2_S production occurs on MB supplemented with sodium thiosulfate, cysteine and sodium sulfate. Acid and alkaline phosphatase, *α*-chymotrypsin, cystine arylamidase, esterase (C4), esterase lipase (C8), leucine arylamidase, naphthol-AS-BI-phosphohydrolase, trypsin and valine arylamidase activities are present. Acid is not produced from citrate, D-cellobiose, D-fructose, D-galactose, D-glucose, D-maltose, D-mannose, D-salicin, D-trehalose, D-xylose, L-arabinose, L-malate, L-glutamic acid, and sucrose. The principal fatty acids are iso-C_15:0_, iso-C_17:0_ 3-OH, iso-C_15:1_ G, and summed feature 3 (C_16:1_*ω*7*c* and/or C_16:1_*ω*6*c*). The sole respiratory quinone is menaquinone-6 (MK-6). The major polar lipids are phosphatidylethanolamine, one unidentified aminophospholipid, one unidentified aminolipid, and one unidentified lipid. In addition, moderate to minor amounts of two unidentified aminoglycolipids, one unidentified glycolipid, and two unidentified lipids are present. The DNA G + C content is 41.0% (by genome).

The type strain A3-108^T^ (CGMCC 1.18821^T^ = KCTC 82563^T^) is isolated from the seawater, collected from the West Pacific Ocean (at depth of 300 m, 23.2°N, 162.3°E). The GenBank/EMBL/DDBJ accession number for the 16S rRNA gene sequence of strain A3-108^T^ is MW244395 and the GenBank accession number for the whole genome sequence is JAECMS000000000.

## Supplementary Information

Below is the link to the electronic supplementary material.

Supplementary file1 (DOCX 3003 KB)

## Data Availability

The GenBank/EMBL/DDBJ accession number for the 16S rRNA gene sequence of strain A3-108^T^ is MW244395. The GenBank accession numbers for the whole genome sequence of strain A3-108^T^ and *Galbibacter mesophilus* CGMCC 1.15663^T^ are JAECMS000000000 and JAERQH000000000, respectively.

## Abbreviations

AAI: Average amino acid identity
ANI: Average nucleotide identity
ANIb: Average nucleotide identity based on BLAST
DDH: DNA–DNA hybridization
MA: Marine agar 2216
MB: Marine broth 2216
MOF medium: Marine oxidation–fermentation medium
ML: Maximum-likelihood
MP: Maximum parsimony
NJ: Neighbor-joining
MK-6: Menaquinone-6
APL: Aminophospholipid
AGL: Aminoglycolipid
PE: Phosphatidylethanolamine
AL: Unidentified aminolipid
GL: Unidentified glycolipid
L: Unidentified lipid
