# Supplementary material for: Luteirhabdus pelagi gen. nov., sp. nov., a novel member of the family Flavobacteriaceae, isolated from the West Pacific Ocean
Source: Arch Microbiol. 2021 Oct 26;203(10):6021–31. doi: 10.1007/s00203-021-02557-3 (PMC8590676; doi:10.1007/s00203-021-02557-3)

**Supplementary materials**

***Luteirhabdus pelagi* gen. nov., sp. nov., a novel member of the family *Flavobacteriaceae*, isolated from the West Pacific Ocean**

Wen-Ting Ren^1,2^, Fan-Xu Meng^1^, Li-Li Guo^1,3^, Li Sun^4^, Xue-Wei Xu^1,2^, Peng Zhou^1,*^, Yue-Hong Wu^1,2,*^

^1^Key Laboratory of Marine Ecosystem Dynamics, Ministry of Natural Resources & Second Institute of Oceanography, Ministry of Natural Resources, Hangzhou 310012, PR China

^2^School of Oceanography, Shanghai Jiao Tong University, Shanghai 200240, PR China

^3^College of Life and Environmental Science, Hunan University of Arts and Science, Changde 415000, PR China

^4^State Research Center of Island Exploitation and Management & Second Institute of Oceanography, Ministry of Natural Resources, Hangzhou 310012, PR China

*Corresponding authors: yuehongwu@sio.org.cn; hockeyextremophiles@yahoo.com.

**Supplementary Table S1** Genomic features of A3-108^T^ and its reference strains.

Strains/species: 1, strain A3-108^T^; 2, *Marinirhabdus gelatinilytica* NH83^T^; 3, *Galbibacter mesophilus* CGMCC 1.15663^T^; 4, *Marixanthomonas ophiurae* JCM 14121^T^.

|  | 1 | 2 | 3 | 4 |
| --- | --- | --- | --- | --- |
| Size (Mb) | 3.40 | 3.29 | 3.77 | 3.30 |
| Contigs | 99 | 24 | 10 | 6 |
| G+C (%) | 41.0 | 41.3 | 37.3 | 35.9 |
| Coding genes | 3250 | 3146 | 3362 | 3097 |
| Protein | 3208 | 3111 | 3318 | 3060 |
| rRNA | 5 | 2 | 3 | 1 |
| tRNA | 37 | 33 | 41 | 36 |
| Accession | JAECMS01 | QRAO01 | JAERQH01 | QVID01 |

**Supplementary** **Table S2** The ANI, DDH and AAI values among the genomes of strain A3-108^T^ with the reference strains.

|  | ANIb | OrthoANI | *in silico* DDH | AAI | Accession number |
| --- | --- | --- | --- | --- | --- |
| 1, Strain A3-108^T^ | / | / | / | / | JAECMS01 |
| 2, *Marinirhabdus gelatinilytica* NH83^T^ | 69.5 | 70.1 | 22.1 | 68.0 | QRAO01 |
| 3, *Galbibacter mesophilus* CGMCC 1.15663^T^ | 67.3 | 67.7 | 18.7 | 58.8 | JAERQH01 |
| 4, *Marixanthomonas ophiurae* JCM 14121^T^ | 71.1 | 71.5 | 18.9 | 71.4 | QVID01 |

**Supplementary Table S3** The biochemical identification of API ZYM and API 20NE of strain A3-108^T^. +, positive; -, negative.

| **API ZYM** | **Result** |
| --- | --- |
| Acid phosphatase, alkaline phosphatase, *α*-chymotrypsin, cystine arylamidase, esterase (C4), esterase lipase (C8), leucine arylamidase, naphthol-AS-BI-phosphohydrolase, trypsin and valine arylamidase | + |
| *N*-acetyl-*β*-glucosaminidase, *β*-fucosidase, *α*-galactosidase, *β*-galactosidase, *α*-glucosidase, *β*-glucosidase, *β*-glucuronidase, lipase (C14), *α*-mannosidase | - |
| **API 20NE** |  |
| Arginine dihydrolase, hydrolysis of gelatin (protease) | + |
| Assimilation of adipic acid, capric acid, D-glucose, D-maltose, D-mannitol, D-mannose, fermentation (glucose), L-arabinose, malate, N-acetyl-glucosamine, phenylacetic acid, potassium gluconate and trisodium citrate, hydrolysis of esculin (*β*-glucosidase), indole production (tryptophane), reduction of nitrate to nitrite, urease, *β*-galactosidase (para-nitrophenyl *β*-D-galactpyranosidase) | - |

**Supplementary Fig. S1.** Transmission electron micrographs showing the cell morphology and ultrastructure of strain A3-108^T^ (a and b). Bars, 0.2 μm (a) and 0.5 μm (b).


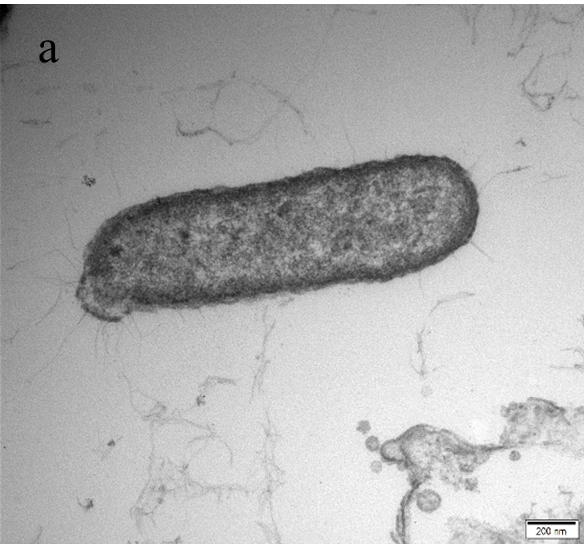

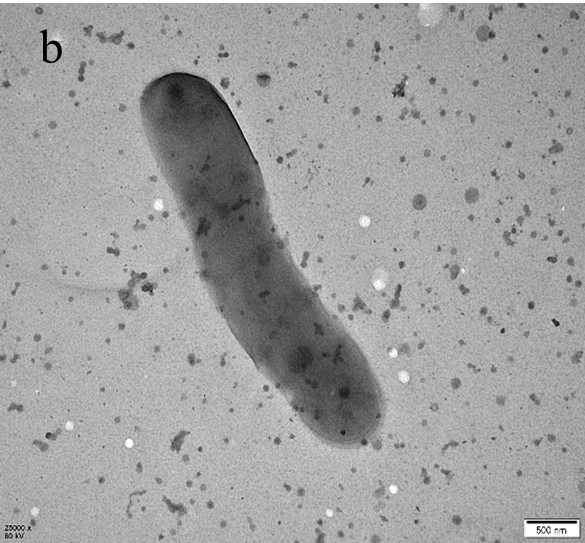


**Supplementary Fig. S2.** The growth curve of strain A3-18^T^ (a) and the curve of log_10_(OD_600_) (b), showing that the lag phase, the exponential and the stationary phases were 0-16 h, 16-40 h and exceed 40 h, respectively. On the basis of formulas, the doubling time and specific growth rate for strain A3-108^T^ were 6.2 h and 0.16 h^-1^, respectively. Doubling time (*t_d_*/h) = ln2/*k*; *k* represents relative growth rate (slope of the curve); specific growth rate (*μ*/h^-1^) = 1/*t_d_*.


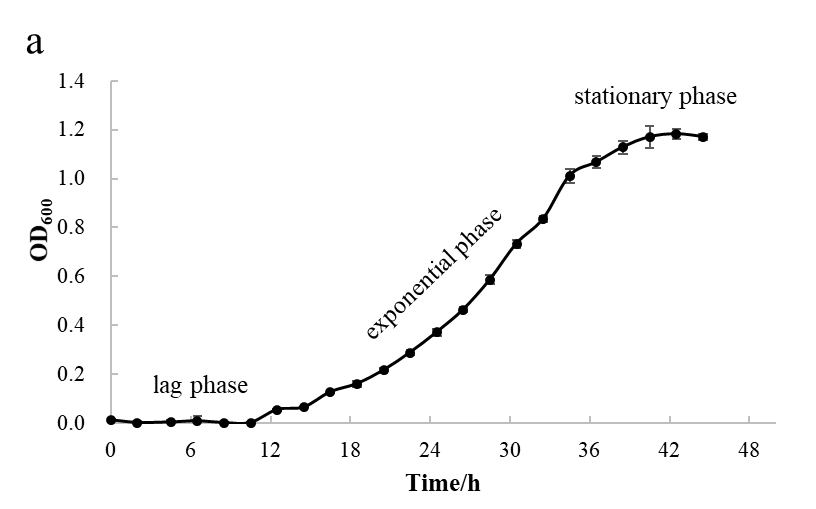

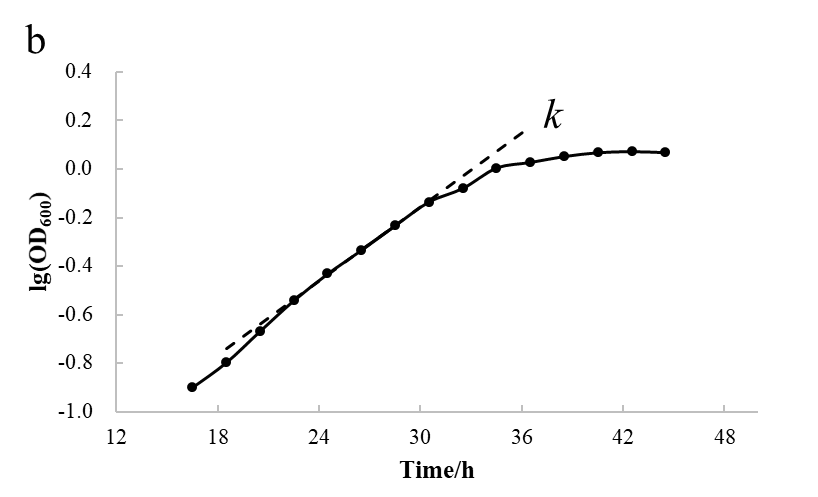


**Supplementary Fig. S3.** Absorption profiles of carotenoids produced by strain A3-108^T^. UV-visible absorption spectrum of crude extract indicate strain A3-108^T^ comprised carotenoids through typical absorption peaks around 470 nm detection wavelength.


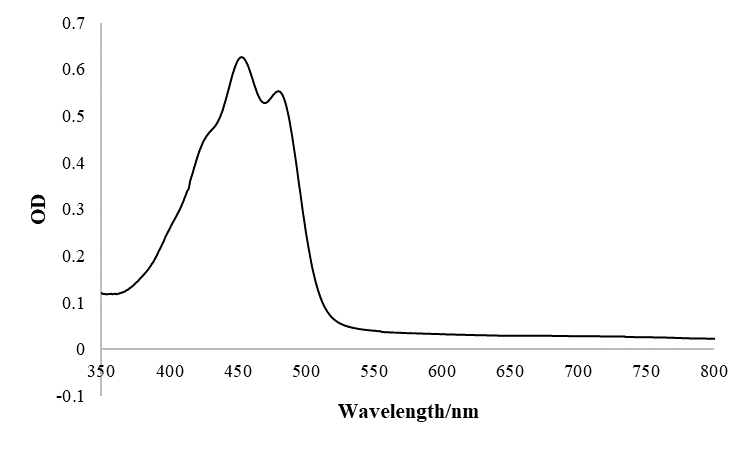


**Supplementary Fig. S4.** Thin-layer chromatograms of strain A3-108^T^ after staining with molybdatophosphoric acid, molybdenum blue reagent, ninhydrin reagent and α-naphthol reagent (a-d). APL, aminophospholipid; AGL, aminoglycolipid; PE, phosphatidylethanolamine; AL, aminolipid; GL, glycolipid; L, lipid.


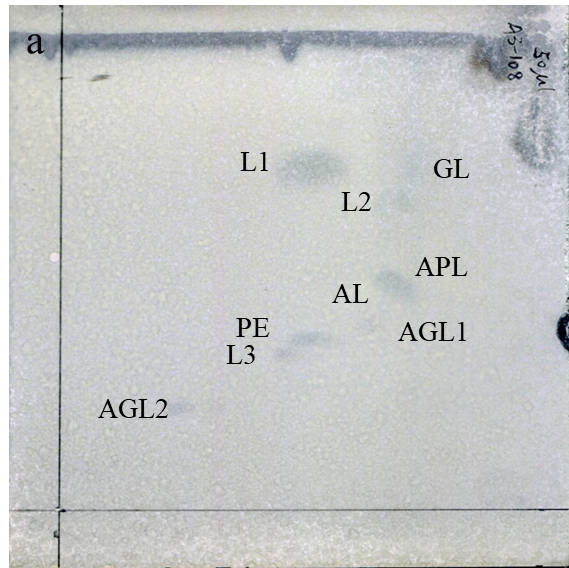

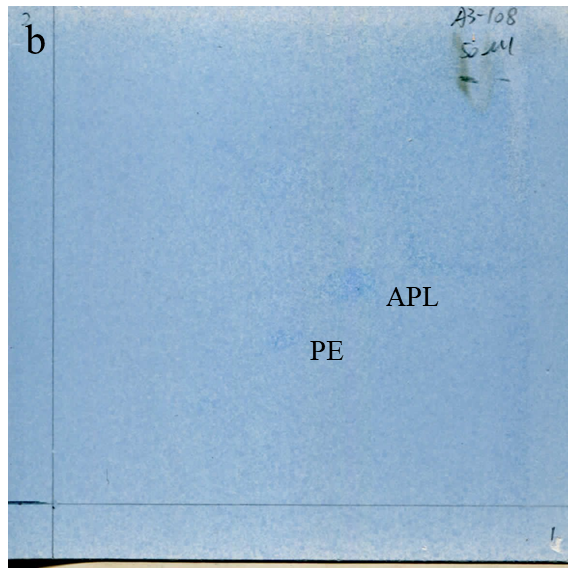

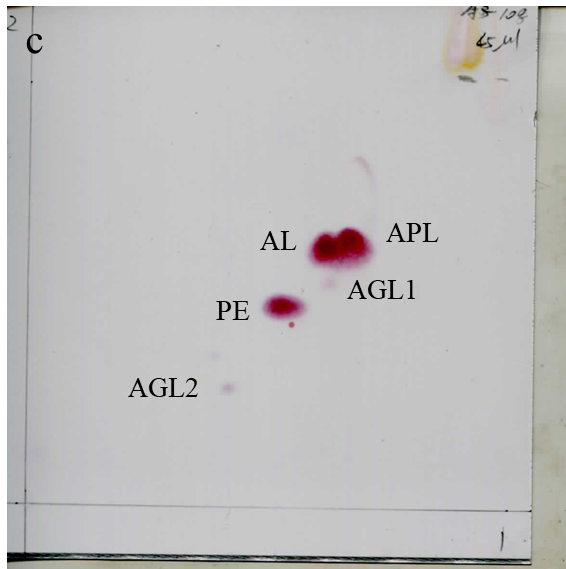

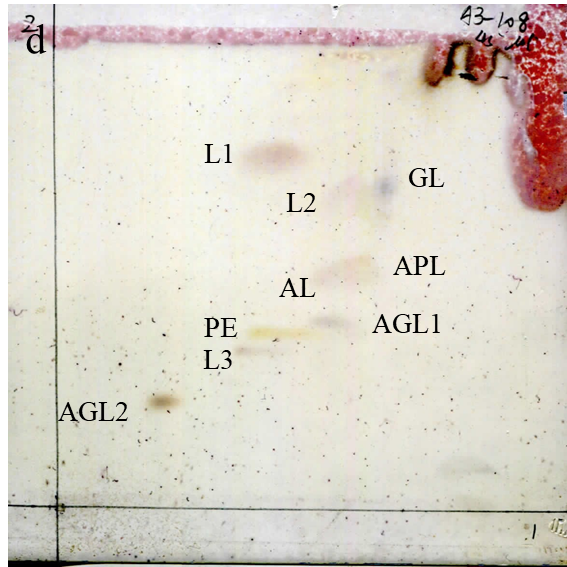

Supplement: Supplementary file 1 — Supplementary file1 (DOCX 3003 KB) [file 203_2021_2557_MOESM1_ESM.docx]
